# A Systematic Review and Meta-Analysis of the Association between Uric Acid and Allantoin and Rheumatoid Arthritis

**DOI:** 10.3390/antiox12081569

**Published:** 2023-08-05

**Authors:** Angelo Zinellu, Arduino A. Mangoni

**Affiliations:** 1 Department of Biomedical Sciences, University of Sassari, 07100 Sassari, Italy; azinellu@uniss.it; 2 Discipline of Clinical Pharmacology, College of Medicine and Public Health, Flinders University, Bedford Park, SA 5042, Australia; 3 Department of Clinical Pharmacology, Flinders Medical Centre, Southern Adelaide Local Health Network, Bedford Park, SA 5042, Australia

**Keywords:** allantoin, uric acid, rheumatoid arthritis, oxidative stress, cardiovascular disease, atherosclerosis

## Abstract

Alterations in the circulating concentrations of uric acid and its degradation product, allantoin, might account for the systemic pro-oxidant state and the increased cardiovascular risk in rheumatoid arthritis (RA). We sought to address this issue by conducting a systematic review and meta-analysis of the association between the plasma/serum concentrations of uric acid and allantoin and RA. We searched PubMed, Scopus, and Web of Science from inception to 20 June 2023 for studies comparing plasma/serum concentrations of uric acid and allantoin between RA patients and healthy controls. We assessed the risk of bias with the JBI Critical Appraisal Checklist for analytical studies and the certainty of evidence with the Grades of Recommendation, Assessment, Development and Evaluation (GRADE) Working Group system. In the 19 studies selected for analysis, there were non-significant differences in uric acid concentrations between RA patients and controls (standard mean difference, SMD = 0.11, 95% CI −0.07 to 0.30, *p* = 0.22; I^2^ = 87.9%, *p* < 0.001; low certainty of evidence). By contrast, the concentrations of allantoin were significantly higher in RA patients (SMD = 1.10, 95% CI 0.66 to 1.55, *p* < 0.001; I^2^ = 55.6%, *p* = 0.08; extremely low certainty of evidence). In meta-regression, a significant association was observed between the SMD of uric acid concentrations and body mass index, a risk factor for atherosclerosis and cardiovascular disease (t = 3.35, *p* = 0.007). Our study has shown a significant increase in the concentrations of the oxidative stress biomarker allantoin in patients with RA. Further research is warranted to investigate the interplay between uric acid, allantoin, redox balance, and cardiovascular disease in this group. (PROSPERO registration number: CRD42023441127).

## 1. Introduction

Patients with rheumatoid arthritis (RA), a chronic and disabling autoimmune condition that is characterized by joint damage, pain, stiffness, and inflammation, often suffer from extra-articular clinical manifestations affecting various organs and tissues [1,2]. Among these extra-articular manifestations, atherosclerosis and cardiovascular disease are particularly common and, importantly, represent the main cause of death in RA patients [3,4,5,6,7]. It has been suggested that the cardiovascular risk imparted by RA is similar in magnitude to diabetes, a major cardiovascular risk factor and a leading cause of mortality and disability worldwide [8]. For example, in an observational study comparing the incidence of cardiovascular disease at three years in three groups—patients with RA, patients with diabetes, and the general population without diabetes and RA—the hazard ratio of cardiovascular disease for RA patients vs. the general population was 1.94 (95% CI 1.24 to 1.95, *p* = 0.004). This was similar to the hazard ratio of diabetic patients vs. the general non-diabetic population, which was 2.04 (95% CI 1.12 to 3.67, *p* = 0.019) [9]. Several structural and functional abnormalities in the cardiovascular system have been reported in patients with RA. These abnormalities primarily involve the reduced synthesis of nitric oxide by endothelial nitric oxide synthase with consequent development of endothelial dysfunction and impaired vasodilation [10,11,12,13,14], intima-media thickening [15], increased arterial stiffness [16,17], and accelerated onset and progression of atherosclerosis [18,19]. These phenomena, also known under the umbrella term “vascular remodeling”, are also responsible for an increase in arterial wave reflection and cardiac afterload which, in turn, favor the development of additional myocardial abnormalities with arrhythmogenic potential in RA patients [20,21]. These alterations explain the excessive risk of ischemic heart disease [14,22], atrial fibrillation [23], stroke [24], and sudden cardiac death in this patient group [25]. Notably, the pathophysiology of RA and the described cardiovascular abnormalities are both characterized by the presence of an impaired redox balance which favors the development of a local and systemic pro-oxidant state [26,27,28,29,30,31,32,33,34]. These observations suggest that the identification of robust and easily measurable biomarkers of oxidative stress might be useful not only to assess RA activity but also the extra-articular cardiovascular manifestations and, potentially, the benefits of specific therapies targeting RA and cardiovascular risk.

**Figure 1:**
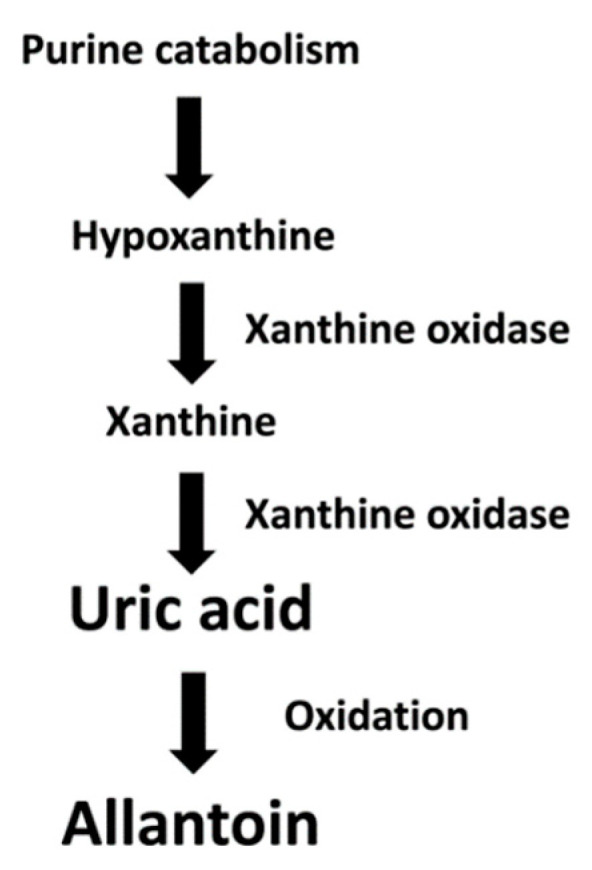
Uric acid metabolic pathways in humans.

Several biomarkers of oxidative stress have been investigated in clinical studies of RA, including markers of lipid oxidation (thiobarbituric and reactive substances, malondialdehyde, and F2-isoprostane), protein oxidation (protein carbonyls, advanced oxidation protein products, and S-nitrosothiols), DNA oxidation (DNA strand breaks, micronucleus), specific enzymes involved in the regulation of redox balance (superoxide dismutase, glutathione peroxidase, myeloperoxidase, catalase, nicotinamide adenine dinucleotide phosphate oxidase, glutathione reductase, arylesterase, and paraoxonase), antioxidant molecules (glutathione, total antioxidant capacity, beta-carotene, vitamin E, thiol group), and free radicals [35,36,37]. However, the analytical challenges that are often encountered with their measurement in various biological samples have so far curtailed the utility of assessing biomarkers of oxidative stress in clinical studies investigating RA and cardiovascular endpoints [38,39,40]. An alternative approach in the search for biomarkers of oxidative stress consists in investigating the biological and pathophysiological role of routinely measured molecules and metabolites that may also affect the redox state, e.g., uric acid and allantoin. Uric acid, routinely measured in clinical practice to monitor patients at risk of gout, is the final product of the metabolism of purine nucleotides and has been shown to exert both antioxidant and pro-oxidant effects [41,42,43]. Allantoin, derived from the non-enzymatic oxidation of uric acid by reactive oxygen species, has also been proposed as a biomarker of oxidative stress in humans (Figure 1) [44,45,46,47].

Notably, there is also robust evidence showing that an increase in serum/plasma concentrations of uric acid is associated with an increased risk of atherosclerotic cardiovascular disease in various patient groups [48,49,50]. Significant associations between uric acid and cardiovascular disease have also been reported in RA [51,52,53], suggesting that the combined assessment of uric acid and allantoin might be useful in investigating alterations in the redox balance as well as extra-articular cardiovascular manifestations in RA patients.

Therefore, we investigated the pathophysiological role of uric acid and allantoin in RA by means of systematic review and meta-analysis and also assessed possible associations between the effect size and a range of clinical and demographic characteristics, including surrogate markers of cardiovascular disease.

## 2. Methods

### 2.1. Systematic Literature Search

We searched Scopus, PubMed, and Web of Science from inception to 20 June 2023, using the following terms: “rheumatoid arthritis” AND “uric acid” OR “allantoin”. Two investigators independently screened the abstracts and, if relevant, the full-text articles according to the following inclusion criteria: (a) the assessment of uric acid and/or allantoin concentrations in plasma or serum; (b) the comparison of RA patients and healthy controls in case-control studies; and (c) the availability of full-text articles in the English language. Additional studies were searched for in the references of individual articles. Any disagreement was resolved by a third investigator.

The following parameters were collected and transferred to an electronic spreadsheet for further analysis: first author details, age and sex distribution of the study participants, year of publication, study continent, sample size, uric acid and allantoin concentrations in serum or plasma, body mass index, lipid profile, and history of hypertension, diabetes, and dyslipidaemia. The risk of bias and the certainty of evidence were assessed using conventional methods, i.e., the JBI checklist and the Grading of Recommendations, Assessment, Development, and Evaluations [54,55]. The study results are presented according to the Preferred Reporting Items for Systematic reviews and Meta-Analyses (PRISMA) 2020 statement (Supplementary Tables S1 and S2) [55]. The review was registered in the International Prospective Register of Systematic Reviews (PROSPERO registration number: CRD42023441127).

### 2.2. Statistical Analysis

We created forest plots using standardized mean differences (SMDs) and 95% confidence intervals (CIs) to assess for differences in the continuous data of uric acid and allantoin concentrations between RA patients and healthy controls (*p*-value < 0.05 for significance). We extrapolated, if necessary, the means and standard deviations from the medians and the interquartile ranges reported in studies [56], and assessed the heterogeneity of the SMD using the Q statistic (significance level set at a *p*-value < 0.10) and the I^2^ statistic [57,58]. Sensitivity analysis was conducted for studies on uric acid and allantoin to confirm the stability of the results [59]. Publication bias was assessed using the Begg’s and the Egger’s tests (a *p*-value of less than 0.05 was considered significant) [60,61], and the “trim-and-fill” method [62]. We also investigated, in univariate meta-regression and subgroup analyses, associations between the effect size and the following parameters: age, male-to-female ratio, year of publication, study continent, sample size, body mass index, lipid profile, and history of hypertension, diabetes, and dyslipidaemia. Statistical analyses were performed using Stata 14 (STATA Corp., College Station, TX, USA).

## 3. Results

### 3.1. Systematic Search

**Figure 2:**
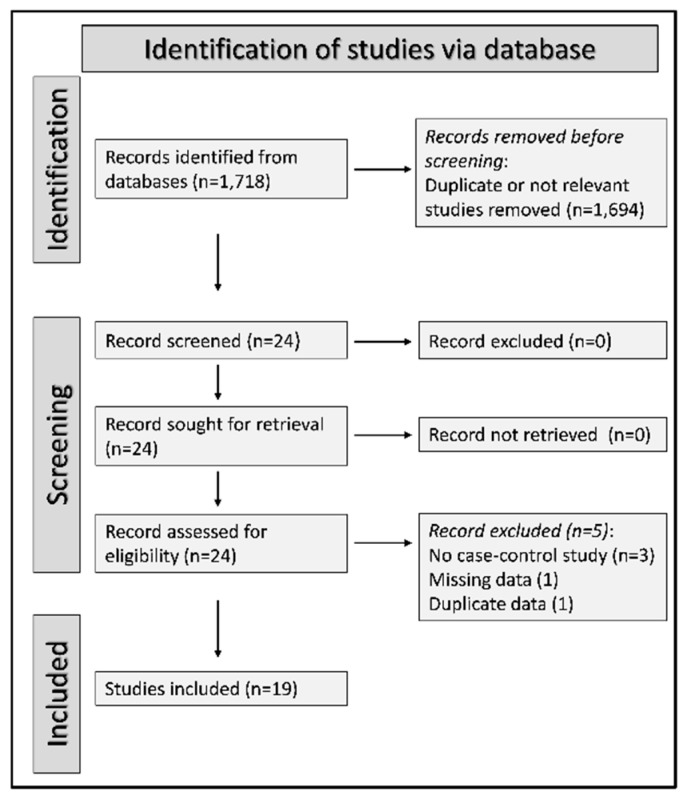
PRISMA 2020 flow diagram describing the study identification, screening, and final selection.

A flow chart describing the study selection is described in Figure 2. There was no disagreement between the two independent investigators, therefore input from a third investigator was not required. We initially identified 1718 articles. A total amount of 1694 was excluded after the initial screening because of duplication or irrelevance to the search question. After reviewing the full text of the remaining 24 articles, a further five were removed because they failed to meet the inclusion criteria, leaving 19 studies, published between 1987 and 2022, for analysis (Table 1 and Table S3) [63,64,65,66,67,68,69,70,71,72,73,74,75,76,77,78,79,80,81].

### 3.2. Uric Acid

Seventeen studies (18 study groups) reported uric acid concentrations in 2845 RA patients (mean age 57 years, 82.4% females) and 1889 healthy controls (mean age 57 years, 77.5% females) [63,64,65,66,69,70,71,72,73,74,75,76,77,78,79,80,81]. Eleven studies were conducted in Asia [64,66,70,71,73,74,77,78,79,80,81], four in Europe [63,65,69,76], and two in America [72,75]. Uric acid was measured in plasma in five studies [63,65,69,72,79], and in serum in the remaining 12 [64,66,70,71,73,74,75,76,77,78,80,81]. The risk of bias was low in all studies, barring two studies with moderate risk (Supplementary Table S4) [63,64]. The initial certainty of evidence was also low given the cross-sectional study design (rating 2, ⊕⊕⊖⊖).

**Figure 3:**
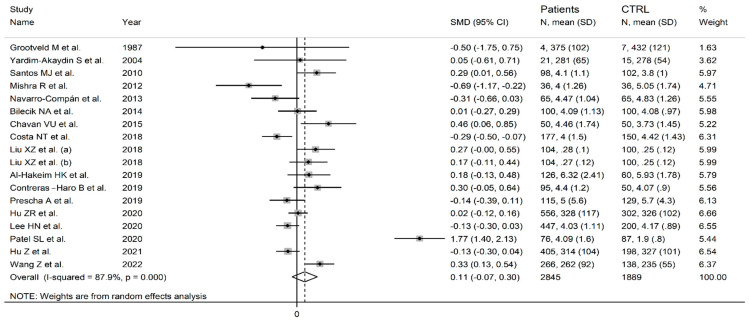
Forest plot of studies reporting uric acid concentrations in RA patients and healthy controls [63,64,65,66,69,70,71,72,73,74,75,76,77,78,79,80,81].

**Figure 4:**
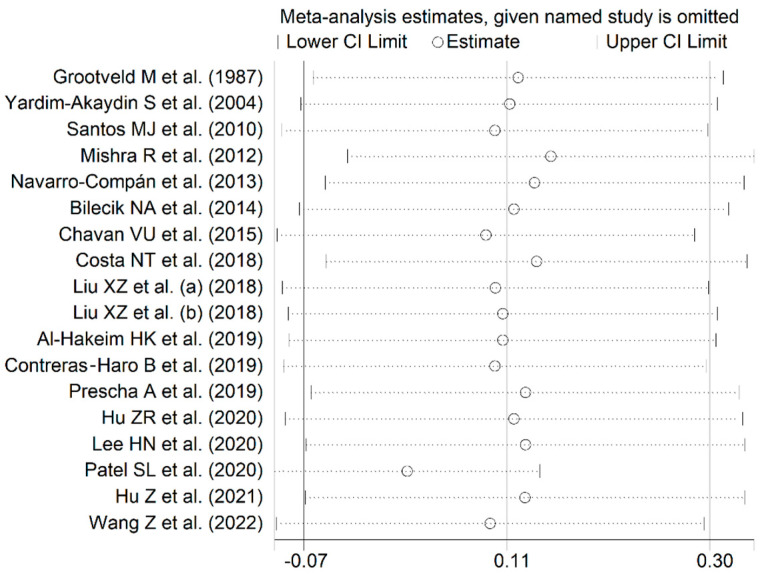
Sensitivity analysis of the association between uric acid and RA [63,64,65,66,69,70,71,72,73,74,75,76,77,78,79,80,81].

**Figure 5:**
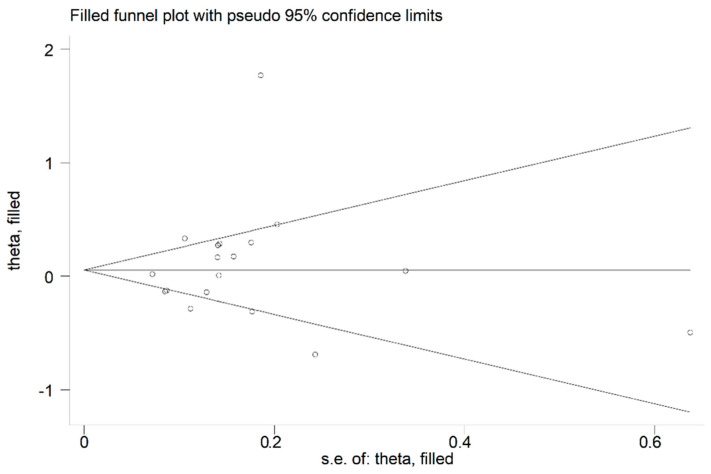
Assessment of publication bias using the funnel plot.

The forest plot shows that the concentrations of uric acid were non-significantly different between RA patients and controls (SMD = 0.11, 95% CI −0.07 to 0.30, *p* = 0.22; I^2^ = 87.9%, *p* < 0.001; Figure 3). Sensitivity analysis showed that the corresponding pooled SMD values were stable (range between 0.02 and 0.15; Figure 4). There was no publication bias according to the Begg’s test (*p* = 0.41), the Egger’s test (*p* = 0.40), or the “trim-and-fill” method (Figure 5).

In meta-regression, non-significant associations were observed between the effect size and age (t = 0.14, *p* = 0.89), sex (t = −1.12, *p* = 0.29), publication year (t = 1.19, *p* = 0.25), and sample size (t = −0.49, *p* = 0.63). Conversely, we observed a significant association between the SMD of uric acid concentrations and the body mass index patients/body mass index controls ratio (t = 3.35, *p* = 0.007; Supplementary Figure S1). Meta-regression investigating associations with lipid profile and a history of hypertension, diabetes, or dyslipidaemia could not be conducted because of the small number of studies reporting these variables (Supplementary Table S3). In subgroup analysis, a non-significant difference (*p* = 0.42) was observed between Asian (SMD = 0.19, 95% CI −0.05 to 0.43, *p* = 0.11; I^2^ = 90.6%, *p* < 0.001) European (SMD = −0.07, 95% CI −0.39 to 0.24, *p* = 0.66; I^2^ = 65.7%, *p* = 0.033), and American studies (SMD = −0.01, 95% CI −0.58 to 0.56, *p* = 0.97; I^2^ = 87.3%, *p* = 0.005), with a lower heterogeneity in European studies (Supplementary Figure S2). Similarly, there were non-significant differences (*p* = 0.45) in pooled SMD between studies assessing serum (SMD = 0.06, 95% CI −0.07 to 0.18, *p* = 0.37; I^2^ = 67.8%, *p* < 0.001) and plasma uric acid concentrations (SMD = 0.24, 95% CI −0.53 to 1.01, *p* = 0.55; I^2^ = 96.0%; *p* < 0.001), with a lower heterogeneity in the former subgroup (Supplementary Figure S3).

The level of certainty remained low (rating 2, ⊕⊕⊖⊖) after considering the low risk of bias in most studies, the high but partly explainable heterogeneity, the lack of indirectness, and the absence of publication bias.

### 3.3. Allantoin

Four studies reported allantoin concentrations in a total of 145 RA patients and 177 healthy controls [63,64,67,68]. Two studies were conducted in Europe [63,67], one in Asia [64], and the remaining one in Oceania [68]. Three studies investigated allantoin in plasma [63,67,68], and the remaining one in serum [64]. Three studies had a moderate risk of bias [63,64,67], and the remaining one a high risk (Supplementary Table S4) [68]. The initial certainty of evidence was low given the cross-sectional study design (rating 2, ⊕⊕⊖⊖).

**Figure 6:**
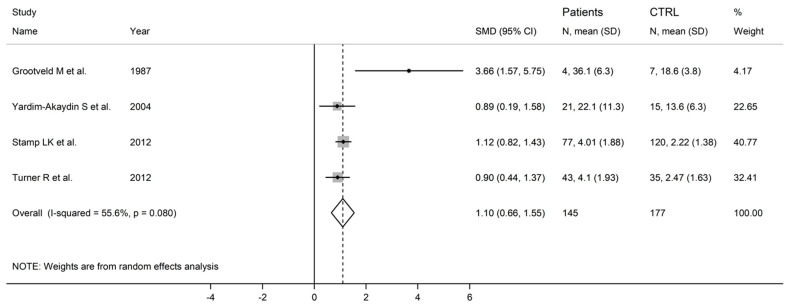
Forest plot of studies reporting allantoin concentrations in RA patients and healthy controls [63,64,67,68].

**Figure 7:**
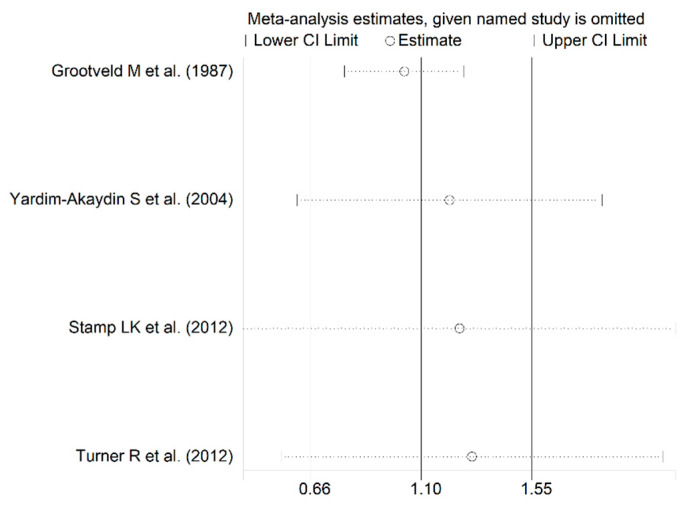
Sensitivity analysis of the association between allantoin and RA [63,64,67,68].

The forest plot showed that the concentrations of allantoin were significantly higher in RA patients compared to controls (SMD = 1.10, 95% CI 0.66 to 1.55, *p* < 0.001; I^2^ = 55.6%, *p* = 0.08; Figure 6). The corresponding pooled SMD values were stable in sensitivity analysis (range between 1.04 and 1.31; Figure 7).

The assessment of publication bias, meta-regression and subgroup analysis could not be conducted because of the limited number of studies selected.

The level of certainty was downgraded to extremely low (rating 0, ⊖⊖⊖⊖) after considering the moderate-high risk of bias (downgrade one level), the large heterogeneity (downgrade one level), the relatively low imprecision (no rating change), the relatively large effect size (SMD = 1.10, upgrade one level), and lack of assessment of publication bias (downgrade one level).

## 4. Discussion

In our study, there were non-significant differences using SMD in plasma/serum concentrations of uric acid between RA patients and healthy controls. By contrast, RA patients had significantly higher plasma/serum allantoin concentrations compared with controls. In meta-regression analysis, a significant association was observed between the SMD of uric acid concentrations and body mass index, a surrogate marker of atherosclerosis and cardiovascular disease. The results of individual studies do not substantially affect the corresponding SMD values in sensitivity analysis. Overall, our results suggest the presence of a pro-oxidant state in RA, as indicated by elevations in circulating allantoin, and a more complex role played by uric acid in terms of redox balance and cardiovascular risk in this patient group.

Uric acid is synthesized in humans primarily in the liver, the intestine, and the endothelium as the end product of exogenous purines and endogenously following the degradation of the purines adenine and guanosine resulting from cell damage or cell death [82]. The precursor xanthine is converted to uric acid by the enzyme xanthine oxidase [83]. In the absence of another enzyme in humans, uricase, uric acid is oxidized to allantoin by reactive oxygen species (Figure 1) [84]. The plasma/serum concentrations of uric acid are the net result of endogenous synthesis, dietary intake, and renal elimination [43]. The biological and pathophysiological role of uric acid in the regulation of the redox state has been investigated in experimental and human studies. Traditionally, uric acid was thought to act predominantly as an antioxidant extracellularly and a pro-oxidant intracellularly [85]. Extracellularly, the antioxidant effects of uric acid primarily involve its interaction with reactive oxygen species, as previously described, to form allantoin [44], and with peroxynitrite to form the compound triuret [86]. However, human studies have provided conflicting results regarding the putative antioxidant effects of uric acid [87,88,89]. It is also important to highlight that uric acid can react with nitric oxide, a key messenger involved in the maintenance of cardiovascular homeostatic mechanisms, to form 6-aminouracil [90]. This reaction reduces the availability of nitric oxide, with a consequent dysregulation of vascular homeostasis [91]. This hypothesis is corroborated by the results of studies showing that a relative increase in uric acid concentrations is associated with an increased risk of hypertension [92], obesity [93], insulin resistance [94], renal disease [95], and cardiovascular disease [96,97]. Significant positive associations between uric acid and mortality have also been reported. For example, in a systematic review and meta-analysis of 24 studies investigating a total of 25,453 patients with chronic kidney disease, those in the upper tertile of uric acid concentrations had a significantly higher risk of death compared with those at the bottom tertile (hazard ratio = 1.52, 95% CI 1.33 to 1.73). In further analyses, each 1mg/dL increase in uric acid concentrations was associated with an 8% increased risk of mortality [98] Similarly, in a prospective study of subjects with diabetes participating in the National Health and Nutrition Examination Survey those in the top quintile of uric acid concentrations had a significantly higher risk of all-cause mortality and cardiovascular mortality compared with those in the bottom quintile (hazard ratio = 1.28, 95% 1.03 to 1.58, and 1.41, 95% CI 1.03 to 1.94, respectively) [97]. Furthermore, uric acid-lowering pharmacological interventions have been shown to exert some beneficial cardiometabolic effects in different patient groups. For example, in a systematic review and meta-analysis of 13 studies the uric acid lowering drug allopurinol reduced systolic blood pressure and diastolic blood pressure to a greater extent than a control group (SMD = 0.32, 95% CI 0.14 to 0.50, *p* < 0.001, and SMD = 0.26, 95% CI 0.10 to 0.42, *p* = 0.001, respectively). Notably, in subgroup analysis the reduction in systolic and diastolic blood pressure was not influenced by the concomitant use of conventional antihypertensive agents [99]. In other systematic reviews and meta-analyses, different pharmacological strategies reducing uric acid concentrations have been shown to reduce the progression of kidney function decline and glucose concentrations, other well established cardiovascular risk factors [100,101]. Intracellularly, uric acid acts as a pro-oxidant by stimulating nicotinamide adenine dinucleotide phosphate oxidases and the peroxynitrite-mediated oxidation of lipids [102,103].

Therefore, the absence of significant differences in uric acid concentrations between RA patients and healthy controls in our systematic review and meta-analysis may reflect the various and sometime opposite effects exerted by this molecule on the redox state and vascular homeostasis. In this context, an interesting observation has been made wherein there is a significant and positive association between the SMD of uric acid concentrations and the ratio between the body mass index of patients and the body mass index of controls. A higher body mass index, a recognized risk factor for cardiovascular disease [104,105], has also been shown to be positively associated with plasma/serum uric acid concentrations in population groups of different age, gender distribution, and ethnicity. For example, in a study investigating 18,473 participants from the National Health and Nutrition Examination Survey there was a significant and independent association between uric acid concentrations and body mass index both in males (β = 1.41, 95% CI 1.32 to 1.50, *p* < 0.0001) and in females (β = 1.85, 95% CI 1.74 to 1.97, *p* < 0.0001). Notably, these associations remained significant in all of the included ethnic groups, i.e., Mexican Americans, other Hispanics, non-Hispanic whites, non-Hispanic blacks, and other groups [106]. In another study, 100 participants without a history of gout received an oral dose of 1.5 g of inosine. At baseline, those with high body mass index had significantly higher serum uric acid concentrations compared with those with low or normal body mass index (0.32 ± 0.08 vs. 0.27 ± 0.07 mmol/L, *p* = 0.0002). Following inosine treatment, the fractional excretion of uric acid was significantly lower in participants with high body mass index, suggesting the presence of significant alterations in the renal capacity of uric acid reabsorption in this group [107]. The results of our meta-regression analysis indicate that a significant association between uric acid and RA is more likely to be observed in RA patients with relatively higher body mass indexes. While this observation is also in line with the putative role of uric acid as a mediating factor in the link between RA and cardiovascular disease, we could not test this hypothesis further as the limited number of selected studies prevented the possibility of further meta-regression and subgroup analysis investigating the association between uric acid and other cardiovascular risk factors (Supplementary Table S3).

The significant increase observed in the circulating concentrations of allantoin further supports the idea of a systemic pro-oxidant state in RA patients and the potential utility of this degradation product of uric acid as a biomarker of oxidative stress, which is facilitated by the significant progress made with the development of robust analytical methods for its determination in biological samples [108,109,110], the limited number of studies identified in our-metanalysis suggests that further research is warranted to confirm the association between allantoin and RA, to investigate the effects of this metabolite on vascular homeostasis and atherosclerosis, and to identify possible treatments affecting its concentrations. In this context, one study investigated the circulating concentrations of allantoin in 50 patients with ischemic heart disease (age 59 ± 11 years, 20% females) and 23 healthy controls (age 50 ± 6 years, 65% females). The concentrations of allantoin were significantly higher in patients with ischemic heart disease (46.5 ± 14.5 vs. 27.2 ± 10.7 μmol/L, *p* < 0.0001). Notably, the between-group differences remained significant after adjusting for age, sex, and serum creatinine concentrations [111]. Another study has recently investigated the effects of treatment with dietary antioxidants on allantoin. In this study, healthy non-smoking participants underwent a randomized controlled cross-over study investigating the effects of a control meal without polyphenol rich foods, and two meals with different doses of polyphenol rich foods. Notably, there was a dose-dependent effect between the consumption of polyphenol rich foods and a significant reduction in post-prandial allantoin concentrations [112].

Our study has several strengths, which include the combined assessment of uric acid and allantoin; the assessment, where possible, of the associations between the effect size of the between-group differences and several clinical and demographic characteristics; and the assessment of the risk of bias and certainty of evidence. By contrast, a significant limitation is represented by the large–extreme heterogeneity observed. However, we identified sources of heterogeneity for uric acid in subgroup analysis (study continent and matrix used for measurement, i.e., plasma vs. serum) and demonstrated that individual studies did not substantially affect the corresponding pooled SMD in sensitivity analysis.

## 5. Conclusions

Our systematic review and meta-analysis have shown that RA patients have similar concentrations of uric acid but significantly higher concentrations of the oxidative stress marker allantoin compared to healthy controls. While caution is needed in the interpretations of our data given the relatively small number of studies identified in our literature search, the results suggest the potential utility of allantoin in monitoring oxidative stress and the need for further research to investigate the complex effects of uric acid on the redox balance and cardiovascular risk in RA. Additional studies should also investigate pharmacological and dietary interventions targeting uric acid and allantoin and their effects on RA disease activity and cardiovascular risk.

## Figures and Tables

**Table 1:** Study characteristics.

	Healthy Controls					Patients with Rheumatoid Arthritis				
Study	n	Age (Years)	M/F	Uric Acid Mean ± SD (mg/dL, µmol/L, or mmol/L)	Allantoin Mean ± SD (µmol/L)	n	Age (Years)	M/F	Uric Acid Mean ± SD (mg/dL, µmol/L, or mmol/L)	Allantoin Mean ± SD (µmol/L)
Grootveld et al., 1987, UK [63]	7	NR	4/3	432 ± 121 *	18.6 ± 3.8	4	NR	2/2	375 ± 102 *	36.1 ± 63
Yardim-Akaydin et al., 2004, Turkey [64]	15	55	3/12	278 ± 54 *	13.6 ± 63	21	55	4/17	281 ± 65 *	22.1 ± 11.3
Santos et al., 2010, Portugal [65]	102	48	0/102	3.8 ± 1.0	NR	98	49	0/98	4.1 ± 1.1	NR
Mishra et al., 2012, India [66]	36	50	11/25	5.05 ± 1.74	NR	36	50	14/22	4.00 ± 1.26	NR
Stamp et al., 2012, UK [67]	120	68	87/33	NR	2.22 ± 1.38	77	55	22/55	NR	4.01 ± 1.88
Turner et al., 2012, New Zealand [68]	35	NR	NR	NR	2.47 ± 1.63	43	NR	NR	NR	4.10 ± 1.93
Navarro-Compan et al., 2013, Spain [69]	65	50	14/51	4.83 ± 1.26	NR	65	50	14/51	4.47 ± 1.04	NR
Bilecik et al., 2014, Turkey [70]	100	51	0/100	4.08 ± 0.97	NR	100	52	0/100	4.09 ± 1.13	NR
Chavan et al., 2015, India [71]	50	NR	20/30	3.73 ± 1.45	NR	50	NR	19/31	4.46 ± 1.74	NR
Costa et al., 2018, Brazil [72]	150	45	42/108	4.42 ± 1.43	NR	177	55	33/144	4.0 ± 1.5	NR
Liu et al., 2018, China [73] (a)	100	62	31/69	0.25 ± 0.12 °	NR	104	62	35/65	0.28 ± 0.10 °	NR
Liu et al., 2018, China [73] (b)	100	62	31/69	0.25 ± 0.12 °	NR	104	64	34/70	0.27 ± 0.12 °	NR
Al-Hakeim et al., 2019, Iraq [74]	60	NR	30/30	5.93 ± 1.78	NR	126	NR	66/60	6.32 ± 2.41	NR
Contreras-Haro et al., 2019, Mexico [75]	50	51	NR	4.07 ± 0.9	NR	95	54	NR	4.4 ± 1.2	NR
Prescha et al., 2019, Poland [76]	129	54	47/82	5.7 ± 4.3	NR	115	52	24/51	5.0 ± 5.6	NR
Hu et al., 2020, China [77]	302	63	73/229	326 ± 102 *	NR	556	58	106/450	328 ± 117 *	NR
Lee et al., 2020, Republic of Korea [78]	200	61	0/200	4.17 ± 0.89	NR	447	61	0/447	4.03 ± 1.11	NR
Patel et al., 2020, India [79]	87	41	29/58	1.9 ± 0.8	NR	76	44	17/59	4.09 ± 1.6	NR
Hu et al., 2021, China [80]	198	60	52/146	327 ± 101 *	NR	405	59	85/317	314 ± 104 *	NR
Wang et al., 2022, China [81]	138	66	26/112	235 ± 55 *	NR	266	55	28/238	262 ± 92 *	NR

Legend: F, female; M, male; NR, not reported; *, µmol/L; °, mmol/L.

## Data Availability

The relevant data are available from A.Z. upon reasonable request.

## Supplementary Materials

The following supporting information can be downloaded at: https://www.mdpi.com/article/10.3390/antiox12081569/s1, Table S1: PRISMA 2020 for abstracts checklist; Table S2: PRISMA 2020 checklist; Table S3: Cardiovascular risk factors in health controls and patients with rheumatoid arthritis. Table S4: The Joanna Briggs Institute critical appraisal checklist; Figure S1: Bubble plot of the univariate meta-regression analysis between the effect size and the body mass index patients/body mass index controls ratio; Figure S2: Forest plot of studies reporting uric acid concentrations in RA patients and controls according to study continent; Figure S3: Forest plot of studies reporting uric acid concentrations in RA patients and controls according to matrix type (serum or plasma).
