# Supplementary material for: A Systematic Review and Meta-Analysis of the Association between Uric Acid and Allantoin and Rheumatoid Arthritis
Source: Antioxidants (Basel). 2023 Aug 5;12(8):1569. doi: 10.3390/antiox12081569 (PMC10451740; doi:10.3390/antiox12081569)
Supplement: Supplementary file 1 [file antioxidants-12-01569-s001.zip › Supplementary_Table_S3.pdf]

Supplementary Table S3. Cardiovascular risk factors in health controls and patients with rheumatoid arthritis.

	Healthy controls			Patients with rheumatoid arthritis		
Study	BMI (kg/m^2)	Tot-Cholesterol<br>LDL-Cholesterol<br>HDL-Cholesterol<br>Triglycerides<br>(mg/dL or mmol/L)	Hypertension<br>Diabetes<br>Dyslipidemia<br>(%)	BMI (kg/m^2)	Tot-Cholesterol<br>LDL-Cholesterol<br>HDL-Cholesterol<br>Triglycerides<br>(mg/dL or mmol/L)	Hypertension<br>Diabetes<br>Dyslipidemia<br>(%)
Grootveld et al, 1987, UK [1]	NR	NR	NR	NR	NR	NR
Yardim-Akaydin et al, 2004, Turkey [2]	NR	NR	NR	NR	NR	NR
Santos et al, 2010, Portugal [3]	NR	207<br>128<br>62<br>98	32<br>6.9<br>56	NR	203<br>123<br>64<br>104	44<br>7.1<br>54
Mishra et al, 2012, India [4]	23	3.45<br>55<br>67<br>86	NR	23	6.56<br>100<br>47<br>143	NR
Stamp et al, 2012, UK [5]	NR	NR	NR	NR	NR	NR
Turner et al, 2012, New Zealand [6]	NR	NR	NR	NR	NR	NR
Navarro-Compan et al, 2013, Spain [7]	26	NR<br>NR<br>57<br>NR	NR	26.3	NR<br>NR<br>52<br>NR	NR
Bilecik et al, 2014, Turkey [8]	27.6	NR<br>NR<br>48<br>129	NR	29	NR<br>NR<br>51<br>121	NR
Chavan et al, 2015, India [9]	NR	196<br>126<br>42<br>137	NR	NR	213<br>151<br>31<br>147	NR
Costa et al, 2018, Brazil [10]	26.2	NR	NR	27.9	NR	NR
Liu et al, 2018, China [11] (a)	23.2	4.45<br>2.37<br>1.50<br>1.12	NR<br>NR<br>NR	24.6	4.56<br>2.71<br>1.24<br>1.80	36.5<br>100<br>28
Liu et al, 2018, China [11] (b)	23.2	4.45<br>2.37<br>1.50<br>1.12	NR	23.2	4.57<br>2.71<br>1.30<br>1.12	NR
Al-Hakeim et al, 2019, Iraq [12]	26.3	NR	NR	25.8	NR	NR
Contreras-Haro et al, 2019, Mexico [13]	29.5	201<br>116<br>48<br>NR	8<br>NR<br>NR	28	197<br>115<br>52<br>NR	33.7<br>NR<br>NR
Prescha et al, 2019, Poland [14]	NR	NR	NR	NR	NR	NR
Hu et al, 2020, China [15]	22.5	NR	NR<br>NR<br>NR	21.9	NR	24.9<br>11.9<br>16.7
Lee et al, 2020, South Korea [16]	23.1	NR	--	22.7	NR	NR
Patel et al, 2020, India [17]	23.6	NR	--	29.1	NR	NR
Hu et al, 2021, China [18]	22.8	NR	37.9<br>17.7<br>29.3	22.1	NR	19<br>11.6<br>13.8
Wang et al, 2022, China [19]	NR	NR	NR	NR	NR	NR

Legend: BMI, body mass index; HDL, high-density lipoprotein; LDL, low-density lipoprotein; NR, not reported.

References

1. Grootveld, M.; Halliwell, B. Measurement of allantoin and uric acid in human body fluids. A potential index of free-radical reactions in vivo? *Biochem J* **1987**, *243*, 803-808, doi:10.1042/bj2430803.
2. Yardim-Akaydin, S.; Sepici, A.; Ozkan, Y.; Torun, M.; Simsek, B.; Sepici, V. Oxidation of uric acid in rheumatoid arthritis: is allantoin a marker of oxidative stress? *Free Radic Res* **2004**, *38*, 623-628, doi:10.1080/10715760410001694044.
3. Santos, M.J.; Vinagre, F.; Silva, J.J.; Gil, V.; Fonseca, J.E. Cardiovascular risk profile in systemic lupus erythematosus and rheumatoid arthritis: a comparative study of female patients. *Acta Reumatol Port* **2010**, *35*, 325-332.
4. Mishra, R.; Singh, A.; Chandra, V.; Negi, M.P.; Tripathy, B.C.; Prakash, J.; Gupta, V. A comparative analysis of serological parameters and oxidative stress in osteoarthritis and rheumatoid arthritis. *Rheumatol Int* **2012**, *32*, 2377-2382, doi:10.1007/s00296-011-1964-1.
5. Stamp, L.K.; Khalilova, I.; Tarr, J.M.; Senthilmohan, R.; Turner, R.; Haigh, R.C.; Winyard, P.G.; Kettle, A.J. Myeloperoxidase and oxidative stress in rheumatoid arthritis. *Rheumatology (Oxford)* **2012**, *51*, 1796-1803, doi:10.1093/rheumatology/kes193.
6. Turner, R.; Stamp, L.K.; Kettle, A.J. Detection of allantoin in clinical samples using hydrophilic liquid chromatography with stable isotope dilution negative ion tandem mass spectrometry. *J Chromatogr B Analyt Technol Biomed Life Sci* **2012**, *891-892*, 85-89, doi:10.1016/j.jchromb.2012.02.009.
7. Navarro-Compan, V.; Melguizo-Madrid, E.; Hernandez-Cruz, B.; Santos-Rey, K.; Leyva-Prado, C.; Gonzalez-Martin, C.; Navarro-Sarabia, F.; Gonzalez-Rodriguez, C. Interaction between oxidative stress and smoking is associated with an increased risk of rheumatoid arthritis: a case-control study. *Rheumatology (Oxford)* **2013**, *52*, 487-493, doi:10.1093/rheumatology/kes286.
8. Bilecik, N.A.; Tuna, S.; Samanci, N.; Balci, N.; Akbas, H. Prevalence of metabolic syndrome in women with rheumatoid arthritis and effective factors. *Int J Clin Exp Med* **2014**, *7*, 2258-2265.
9. Chavan, V.U.; Ramavataram, D.; Patel, P.A.; Rupani, M.P. Evaluation of serum magnesium, lipid profile and various biochemical parameters as risk factors of cardiovascular diseases in patients with rheumatoid arthritis. *J Clin Diagn Res* **2015**, *9*, BC01-05, doi:10.7860/JCDR/2015/12206.5740.
10. Costa, N.T.; Scavuzzi, B.M.; Iriyoda, T.M.V.; Lozovoy, M.A.B.; Alfieri, D.F.; de Medeiros, F.A.; de Sa, M.C.; Micheletti, P.L.; Sekiguchi, B.A.; Reiche, E.M.V.; et al. Metabolic syndrome and the decreased levels of uric acid by leflunomide favor redox imbalance in patients with rheumatoid arthritis. *Clin Exp Med* **2018**, *18*, 363-372, doi:10.1007/s10238-018-0500-y.
11. Liu, X.Z.; Gao, Y.; Fan, J.; Xu, X.; Zhang, J.; Gao, J.; Wan, W.; Zhao, D.B. Metabolic abnormalities in rheumatoid arthritis patients with comorbid diabetes mellitus. *Clin Rheumatol* **2018**, *37*, 219-226, doi:10.1007/s10067-017-3847-7.
12. Al-Hakeim, H.K.; Moustafa, S.R.; Jasem, K.M. Serum Cesium, Rhenium, and Rubidium in Rheumatoid Arthritis Patients. *Biol Trace Elem Res* **2019**, *189*, 379-386, doi:10.1007/s12011-018-1497-5.
13. Contreras-Haro, B.; Hernandez-Gonzalez, S.O.; Gonzalez-Lopez, L.; Espinel-Bermudez, M.C.; Garcia-Benavides, L.; Perez-Guerrero, E.; Vazquez-Villegas, M.L.; Robles-Cervantes, J.A.; Salazar-Paramo, M.; Hernandez-Corona, D.M.; et al. Fasting triglycerides and glucose index: a useful screening test for assessing insulin resistance in patients diagnosed with rheumatoid arthritis and systemic lupus erythematosus. *Diabetol Metab Syndr* **2019**, *11*, 95, doi:10.1186/s13098-019-0495-x.
14. Prescha, A.; Zablocka-Slowinska, K.; Placzkowska, S.; Gorczyca, D.; Luczak, A.; Grajeta, H. Silicon intake and plasma level and their relationships with systemic redox and inflammatory markers in rheumatoid arthritis patients. *Adv Clin Exp Med* **2019**, *28*, 1485-1494, doi:10.17219/acem/105380.

15. Hu, Z.; Xu, S.; Lin, H.; Ni, W.; Yang, Q.; Qi, J.; Du, K.; Gu, J.; Lin, Z. Prevalence and risk factors for bone loss in Southern Chinese with rheumatic diseases. *BMC Musculoskelet Disord* **2020**, *21*, 416, doi:10.1186/s12891-020-03403-1.
16. Lee, H.N.; Kim, A.; Kim, Y.; Kim, G.T.; Sohn, D.H.; Lee, S.G. Higher serum uric acid levels are associated with reduced risk of hip osteoporosis in postmenopausal women with rheumatoid arthritis. *Medicine (Baltimore)* **2020**, *99*, e20633, doi:10.1097/MD.0000000000020633.
17. Patel, S.L.; Prakash, J.; Gupta, V. TGF-beta1 +869C/T polymorphism increases susceptibility to rheumatoid arthritis in North Indian population. *Clin Rheumatol* **2020**, *39*, 2881-2888, doi:10.1007/s10067-020-05064-w.
18. Hu, Z.; Zhang, L.; Lin, Z.; Zhao, C.; Xu, S.; Lin, H.; Zhang, J.; Li, W.; Chu, Y. Prevalence and risk factors for bone loss in rheumatoid arthritis patients from South China: modeled by three methods. *BMC Musculoskelet Disord* **2021**, *22*, 534, doi:10.1186/s12891-021-04403-5.
19. Wang, Z.; Wang, W.; Xiang, T.; Gong, B.; Xie, J. Serum Uric Acid as a Diagnostic Biomarker for Rheumatoid Arthritis-Associated Interstitial Lung Disease. *Inflammation* **2022**, *45*, 1800-1814, doi:10.1007/s10753-022-01661-w.
