# Supplementary material for: A Systematic Review and Meta-Analysis of the Association between Uric Acid and Allantoin and Rheumatoid Arthritis
Source: Antioxidants (Basel). 2023 Aug 5;12(8):1569. doi: 10.3390/antiox12081569 (PMC10451740; doi:10.3390/antiox12081569)
Supplement: Supplementary file 1 [file antioxidants-12-01569-s001.zip › Supplementary_Table_S4.pdf]

Supplementary Table S4. The Joanna Briggs Institute critical appraisal checklist.

Study	Were the criteria for inclusion clearly defined?	Were the subjects and the setting described in detail?	Was the exposure measured in a valid and reliable way?	Were objective, standard criteria used for measurement of the condition?	Were confounding factors identified?	Were strategies to deal with confounding factors stated?	Were the outcomes measured in a valid and reliable way?	Was appropriate statistical analysis used?	Risk of bias
Grootveld et al [1]	No	No	Yes	Yes	No	No	Yes	Yes	Moderate
Yardim-Akaydin et al [2]	No	No	Yes	Yes	No	No	Yes	Yes	Moderate
Santos et al [3]	Yes	Yes	Yes	Yes	Yes	Yes	Yes	Yes	Low
Mishra et al [4]	Yes	Yes	Yes	Yes	No	No	Yes	Yes	Low
Stamp et al [5]	No	No	Yes	Yes	No	No	Yes	Yes	Moderate
Turner et al [6]	No	No	Yes	No	No	No	Yes	Yes	High
Navarro-Compan et al [7]	No	No	Yes	Yes	Yes	Yes	Yes	Yes	Low
Bilecik et al [8]	Yes	Yes	Yes	Yes	No	No	Yes	Yes	Low
Chavan et al [9]	Yes	Yes	Yes	Yes	No	No	Yes	Yes	Low
Costa et al [10]	Yes	Yes	Yes	Yes	Yes	Yes	Yes	Yes	Low
Liu et al [11]	Yes	Yes	Yes	Yes	No	No	Yes	Yes	Low
Al-Hakeim et al [12]	Yes	Yes	Yes	Yes	Yes	Yes	Yes	Yes	Low
Contreras-Haro et al [13]	Yes	Yes	Yes	Yes	Yes	Yes	Yes	Yes	Low
Prescha et al [14]	Yes	Yes	Yes	Yes	No	No	Yes	Yes	Low
Hu et al [15]	Yes	Yes	Yes	No	Yes	Yes	Yes	Yes	Low
Lee et al [16]	Yes	Yes	Yes	Yes	Yes	Yes	Yes	Yes	Low
Patel et al [17]	Yes	Yes	Yes	Yes	No	No	Yes	Yes	Low
Hu et al [18]	Yes	Yes	Yes	Yes	Yes	Yes	Yes	Yes	Low
Wang et al [19]	Yes	Yes	Yes	Yes	Yes	Yes	Yes	Yes	Low

References

1. Grootveld, M.; Halliwell, B. Measurement of allantoin and uric acid in human body fluids. A potential index of free-radical reactions in vivo? *Biochem J* **1987**, *243*, 803-808, doi:10.1042/bj2430803.
2. Yardim-Akaydin, S.; Sepici, A.; Ozkan, Y.; Torun, M.; Simsek, B.; Sepici, V. Oxidation of uric acid in rheumatoid arthritis: is allantoin a marker of oxidative stress? *Free Radic Res* **2004**, *38*, 623-628, doi:10.1080/10715760410001694044.
3. Santos, M.J.; Vinagre, F.; Silva, J.J.; Gil, V.; Fonseca, J.E. Cardiovascular risk profile in systemic lupus erythematosus and rheumatoid arthritis: a comparative study of female patients. *Acta Reumatol Port* **2010**, *35*, 325-332.
4. Mishra, R.; Singh, A.; Chandra, V.; Negi, M.P.; Tripathy, B.C.; Prakash, J.; Gupta, V. A comparative analysis of serological parameters and oxidative stress in osteoarthritis and rheumatoid arthritis. *Rheumatol Int* **2012**, *32*, 2377-2382, doi:10.1007/s00296-011-1964-1.
5. Stamp, L.K.; Khalilova, I.; Tarr, J.M.; Senthilmohan, R.; Turner, R.; Haigh, R.C.; Winyard, P.G.; Kettle, A.J. Myeloperoxidase and oxidative stress in rheumatoid arthritis. *Rheumatology (Oxford)* **2012**, *51*, 1796-1803, doi:10.1093/rheumatology/kes193.
6. Turner, R.; Stamp, L.K.; Kettle, A.J. Detection of allantoin in clinical samples using hydrophilic liquid chromatography with stable isotope dilution negative ion tandem mass spectrometry. *J Chromatogr B Analyt Technol Biomed Life Sci* **2012**, *891-892*, 85-89, doi:10.1016/j.jchromb.2012.02.009.
7. Navarro-Compan, V.; Melguizo-Madrid, E.; Hernandez-Cruz, B.; Santos-Rey, K.; Leyva-Prado, C.; Gonzalez-Martin, C.; Navarro-Sarabia, F.; Gonzalez-Rodriguez, C. Interaction between oxidative stress and smoking is associated with an increased risk of rheumatoid arthritis: a case-control study. *Rheumatology (Oxford)* **2013**, *52*, 487-493, doi:10.1093/rheumatology/kes286.
8. Bilecik, N.A.; Tuna, S.; Samanci, N.; Balci, N.; Akbas, H. Prevalence of metabolic syndrome in women with rheumatoid arthritis and effective factors. *Int J Clin Exp Med* **2014**, *7*, 2258-2265.
9. Chavan, V.U.; Ramavataram, D.; Patel, P.A.; Rupani, M.P. Evaluation of serum magnesium, lipid profile and various biochemical parameters as risk factors of cardiovascular diseases in patients with rheumatoid arthritis. *J Clin Diagn Res* **2015**, *9*, BC01-05, doi:10.7860/JCDR/2015/12206.5740.
10. Costa, N.T.; Scavuzzi, B.M.; Iriyoda, T.M.V.; Lozovoy, M.A.B.; Alfieri, D.F.; de Medeiros, F.A.; de Sa, M.C.; Micheletti, P.L.; Sekiguchi, B.A.; Reiche, E.M.V.; et al. Metabolic syndrome and the decreased levels of uric acid by leflunomide favor redox imbalance in patients with rheumatoid arthritis. *Clin Exp Med* **2018**, *18*, 363-372, doi:10.1007/s10238-018-0500-y.
11. Liu, X.Z.; Gao, Y.; Fan, J.; Xu, X.; Zhang, J.; Gao, J.; Wan, W.; Zhao, D.B. Metabolic abnormalities in rheumatoid arthritis patients with comorbid diabetes mellitus. *Clin Rheumatol* **2018**, *37*, 219-226, doi:10.1007/s10067-017-3847-7.
12. Al-Hakeim, H.K.; Moustafa, S.R.; Jasem, K.M. Serum Cesium, Rhenium, and Rubidium in Rheumatoid Arthritis Patients. *Biol Trace Elem Res* **2019**, *189*, 379-386, doi:10.1007/s12011-018-1497-5.
13. Contreras-Haro, B.; Hernandez-Gonzalez, S.O.; Gonzalez-Lopez, L.; Espinel-Bermudez, M.C.; Garcia-Benavides, L.; Perez-Guerrero, E.; Vazquez-Villegas, M.L.; Robles-Cervantes, J.A.; Salazar-Paramo, M.; Hernandez-Corona, D.M.; et al. Fasting triglycerides and glucose index: a useful screening test for assessing insulin resistance in patients diagnosed with rheumatoid arthritis and systemic lupus erythematosus. *Diabetol Metab Syndr* **2019**, *11*, 95, doi:10.1186/s13098-019-0495-x.
14. Prescha, A.; Zablocka-Slowinska, K.; Placzkowska, S.; Gorczyca, D.; Luczak, A.; Grajeta, H. Silicon intake and plasma level and their relationships with systemic redox and inflammatory markers in rheumatoid arthritis patients. *Adv Clin Exp Med* **2019**, *28*, 1485-1494, doi:10.17219/acem/105380.
15. Hu, Z.; Xu, S.; Lin, H.; Ni, W.; Yang, Q.; Qi, J.; Du, K.; Gu, J.; Lin, Z. Prevalence and risk factors for bone loss in Southern Chinese with rheumatic diseases. *BMC Musculoskelet Disord* **2020**, *21*, 416, doi:10.1186/s12891-020-03403-1.
16. Lee, H.N.; Kim, A.; Kim, Y.; Kim, G.T.; Sohn, D.H.; Lee, S.G. Higher serum uric acid levels are associated with reduced risk of hip osteoporosis in postmenopausal women with rheumatoid arthritis. *Medicine (Baltimore)* **2020**, *99*, e20633, doi:10.1097/MD.0000000000020633.
17. Patel, S.L.; Prakash, J.; Gupta, V. TGF-beta1 +869C/T polymorphism increases susceptibility to rheumatoid arthritis in North Indian population. *Clin Rheumatol* **2020**, *39*, 2881-2888, doi:10.1007/s10067-020-05064-w.

18. Hu, Z.; Zhang, L.; Lin, Z.; Zhao, C.; Xu, S.; Lin, H.; Zhang, J.; Li, W.; Chu, Y. Prevalence and risk factors for bone loss in rheumatoid arthritis patients from South China: modeled by three methods. *BMC Musculoskelet Disord* **2021**, *22*, 534, doi:10.1186/s12891-021-04403-5.
19. Wang, Z.; Wang, W.; Xiang, T.; Gong, B.; Xie, J. Serum Uric Acid as a Diagnostic Biomarker for Rheumatoid Arthritis-Associated Interstitial Lung Disease. *Inflammation* **2022**, *45*, 1800-1814, doi:10.1007/s10753-022-01661-w.
