# Supplementary figures and images for: A Systematic Review and Meta-Analysis of the Association between Uric Acid and Allantoin and Rheumatoid Arthritis
Source: Antioxidants (Basel). 2023 Aug 5;12(8):1569. doi: 10.3390/antiox12081569 (PMC10451740; doi:10.3390/antiox12081569)

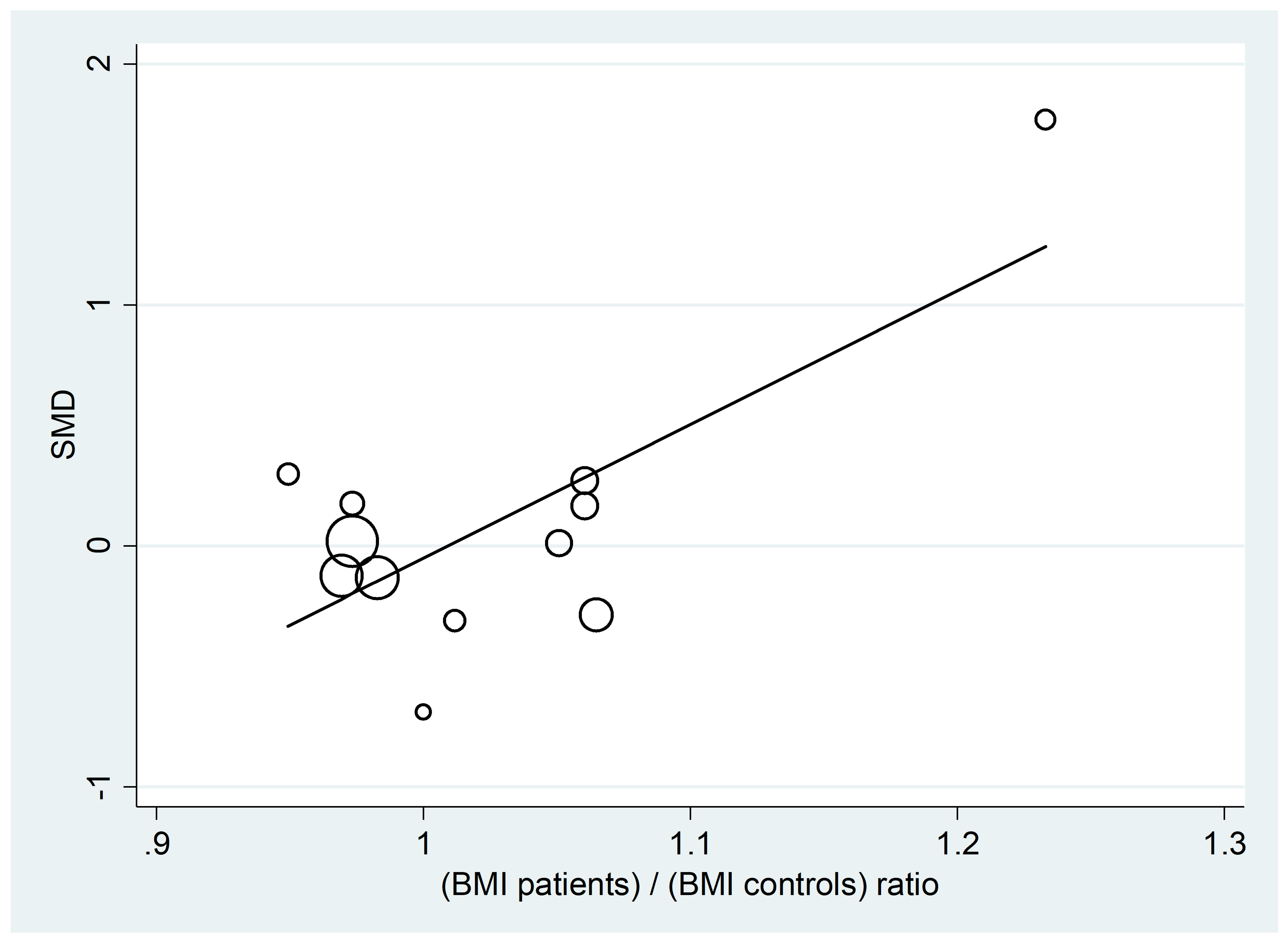

Supplement: Supplementary file 1 [file antioxidants-12-01569-s001.zip › Supplementary_Figure_S1.tif]

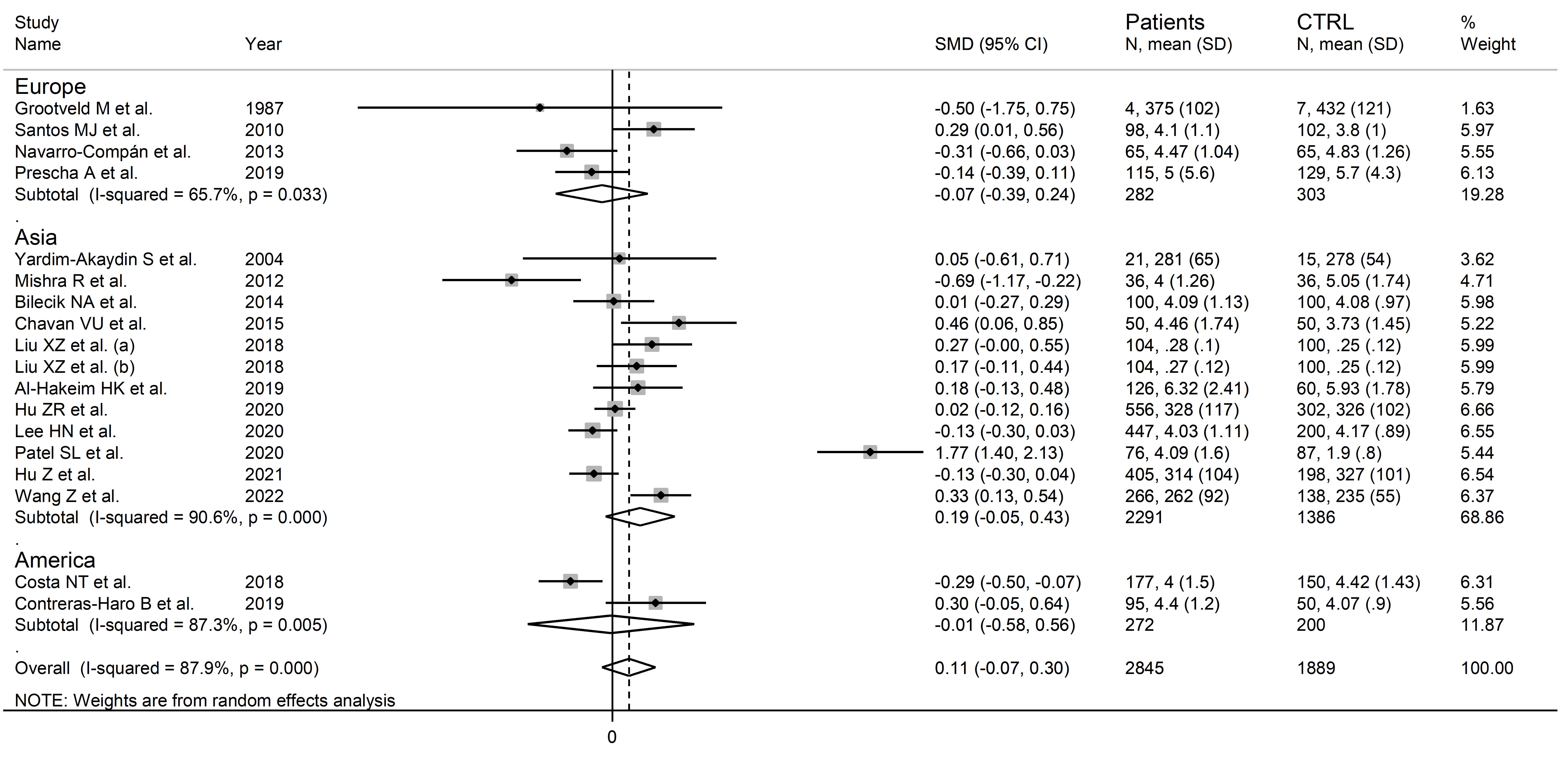

Supplement: Supplementary file 1 [file antioxidants-12-01569-s001.zip › Supplementary_Figure_S2.tif]

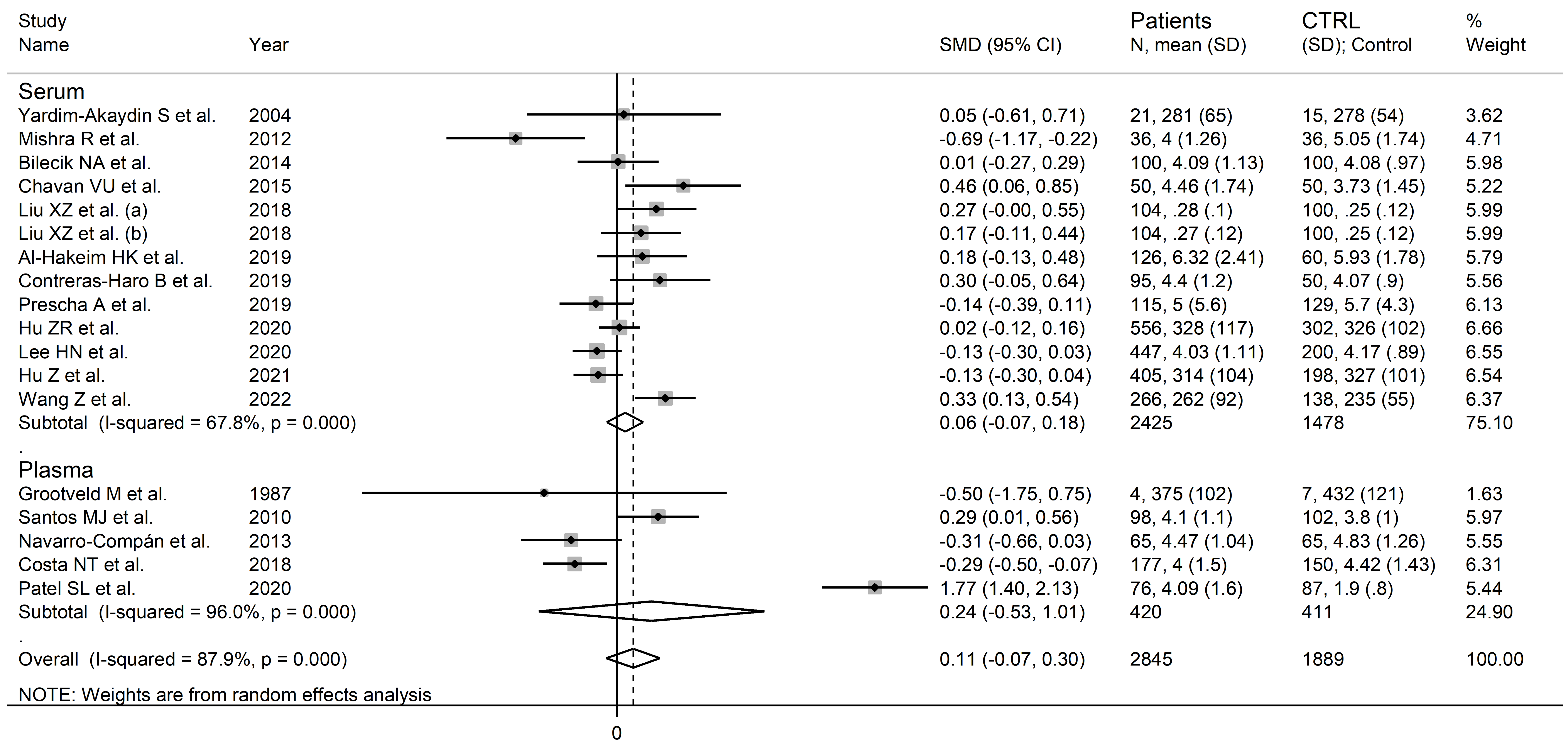

Supplement: Supplementary file 1 [file antioxidants-12-01569-s001.zip › Supplementary_Figure_S3.tif]
